# Identification of Species-Specific MicroRNAs Provides Insights into Dynamic Evolution of MicroRNAs in Plants

**DOI:** 10.3390/ijms232214273

**Published:** 2022-11-17

**Authors:** Zhonglong Guo, Zheng Kuang, Yang Deng, Lei Li, Xiaozeng Yang

**Affiliations:** 1 Co-Innovation Center for Sustainable Forestry in Southern China, College of Biology and the Environment, Nanjing Forestry University, Nanjing 210037, China; 2 Beijing Agro-Biotechnology Research Center, Beijing Academy of Agriculture and Forestry Sciences, Beijing 100097, China; 3 State Key Laboratory of Protein and Plant Gene Research, School of Advanced Agricultural Sciences, Peking University, Beijing 100871, China

**Keywords:** species-specific, miRNA, plant, evolution, metabolism

## Abstract

MicroRNAs (miRNAs) are an important class of regulatory small RNAs that program gene expression, mainly at the post-transcriptional level. Although sporadic examples of species-specific miRNAs (termed SS-miRNAs) have been reported, a genome-scale study across a variety of distant species has not been assessed. Here, by comprehensively analyzing miRNAs in 81 plant species phylogenetically ranging from chlorophytes to angiosperms, we identified 8048 species-specific miRNAs from 5499 families, representing over 61.2% of the miRNA families in the examined species. An analysis of the conservation from different taxonomic levels supported the high turnover rate of SS-miRNAs, even over short evolutionary distances. A comparison of the intrinsic features between SS-miRNAs and NSS-miRNAs (non-species-specific miRNAs) indicated that the AU content of mature miRNAs was the most striking difference. Our data further illustrated a significant bias of the genomic coordinates towards SS-miRNAs lying close to or within genes. By analyzing the 125,267 putative target genes for the 7966 miRNAs, we found the preferentially regulated functions of SS-miRNAs related to diverse metabolic processes. Collectively, these findings underscore the dynamic evolution of miRNAs in the species-specific lineages.

## 1. Introduction

One of the most exciting findings in the recent history of molecular biology is the discovery of the diverse roles of small RNAs in regulating organismal functions [1,2,3,4,5,6]. In particular, microRNAs (miRNAs) constitute an important class of 20–24-nucleotide (nt) small RNAs in eukaryotes [7,8,9,10]. In plants, miRNAs arise from primary transcripts called pri-miRNAs that are generally transcribed by RNA polymerase II as individual transcription units [8,9,11]. The immediate miRNA precursors, pre-miRNAs, contain sequences that form the characteristic intramolecular hairpin structures, thereby abrogating the need for an RNA-dependent RNA polymerase to produce the double-stranded intermediates necessary for the biogenesis of other small regulatory RNAs [1,2,12,13]. Processed by evolutionarily conserved cellular machinery (DICER-like in plants), the yielded mature miRNAs guide both transcriptional and post-transcriptional gene regulations by acting in *trans* as repressors [2,8,14].

As the identification and catalogue of miRNAs continues in an increasing number of species, recent studies on a large phylogenetic scale have advanced our knowledge on the conservation and evolution of miRNAs in the major lineages of eukaryotic groups [15,16,17]. There are three models of de novo miRNA origination that have been conceptualized in both plants and animals. Miniature inverted-repeat transposable elements (MITEs), as the truncated derivatives of autonomous DNA transposons, have been proven to generate miRNAs based on the terminal inverted repeats located at both ends of the MITEs to produce the imperfect hairpin structures [18,19,20,21]. More recently, a genome-scale study including 22 representative plants concluded that the predominant miRNAs in angiosperms were derived from MITEs. The long terminal repeat (LTR) model suggested that retrotransposons connected in opposite directions could process transcripts produced from readthrough events. These LTR-containing transcripts initially fold into long hairpins, triggering the formation of siRNAs. Then, some of them might eventually generate miRNAs [2,22]. The third model is the target-gene inverted duplication model, which assumes that occasional inverted duplication owing to gene family expansion might further process transcripts with near-perfect hairpin structures following the production of miRNAs [10,21,23]. Findings in representative species of the phylum Cnidaria also indicated that miRNA precursors might originate from their own target genes, indicating an ancestral mechanism in both plants and early animals [24]. Although most studies concentrated on conserved miRNAs, more and more findings related to species-specific miRNAs (referred to as SS-miRNAs) are intriguing, supporting the hypothesis that some of them have established a regulatory function in governing organ development, apoptosis, responses to stimuli, metabolism, and cell wall remodeling [25,26,27,28,29,30,31]. However, SS-miRNAs are reported by sporadic examples and have not been assessed on the genome scale in a large number of phylogenetically representative plant species. Therefore, a comprehensive survey of the landscape of SS-miRNAs is worth carrying out.

Bioinformatics techniques for the accurate identification of SS-miRNAs generally have two stumbling blocks. First, there is a need for a variety of species with high-quality annotations of miRNAs. Second, frequent variants on the stem-loops of miRNA genes make the routine homology-based method inadequate for detecting SS-miRNAs. In this study, we performed a systemic investigation of SS-miRNAs from 81 plant species phylogenetically ranging from chlorophytes to angiosperms using the seq-based strategy. The overarching results exhibited a landscape containing 8048 SS-miRNAs from 5499 families of plants and demonstrated the high turnover rate of SS-miRNAs, even among the closely related lineages. We further found that the SS-miRNAs lay close to or within genes and preferentially regulated target genes associated with multiple metabolic processes. These findings provide new insights into the dynamic evolution and diverse metabolic adaptations of novel miRNAs in plants.

## 2. Results

### 2.1. Comprehensive Comparison of Homology-Based and Seq-Based Strategies

**Figure 1:**
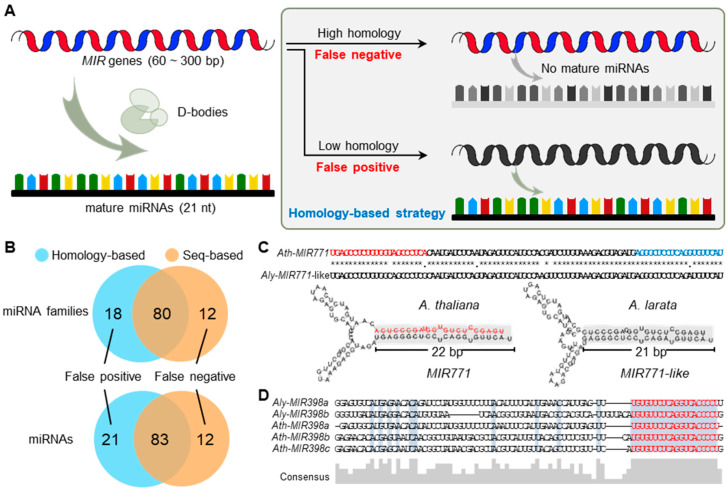
Comparison of homology-based and seq-based strategies for cataloging SS-miRNAs. (**A**) A schematic diagram showing a false negative and a false positive of SS-miRNAs caused by the homology-based strategy. (**B**) A comparison of the results of SS-miRNAs and SS-miRNA families in *A. thaliana* identified using the two strategies. (**C**) Sequence alignments and secondary structures, showing a false negative SS-miRNA family (*MIR771*) mistakenly identified by the homology-based approach. Mature and star miR771 are highlighted in red and blue letters, respectively. The same nucleotides are marked in *. (**D**) Sequence alignments of the *MIR398* family in *A. thaliana* and *A. lyrate*, showing a false positive SS-miRNA family in *A. thaliana*.

Currently, the common approach to study the conservation of miRNAs borrows from the canonical method for studying protein-coding genes, namely searching homologous sequences in other genomes (referred to as the homology-based strategy). However, with the exponential accumulation of sRNA-seq datasets, two glaring drawbacks of the homology-based strategy gradually catch researchers’ eyes (Figure 1A). First, the analysis of sRNA-seq datasets suggests that some of the miRNA candidates identified by the homology-based strategy lack the unique characteristics of miRNAs, which are required to have over 75% of reads corresponding to the mature and star regions [32,33], indicating that many of these are not bona fide miRNAs (Figure 1A). In this case, many true SS-miRNAs are mistakenly considered to be conserved, causing their number to be underestimated (false negative). Second, a growing body of evidence has revealed that the rapid divergence of miRNA hairpins results in low homology among the miRNA loci from an identical miRNA family. Therefore, the homology-based strategy may increase the false positive rate of SS-miRNAs owing to the great variation in hairpins (Figure 1A). These two drawbacks have presented the evolutionary analysis of miRNAs with a puzzle.

Here, we propose a seq-based strategy to identify SS-miRNAs, which has three requirements: (i) no similar mature miRNAs with no more than two mismatches; (ii) hairpin sequences with stable secondary structures, and (iii) hairpins of miRNAs exhibiting the canonical read distribution profile. To prove the improvement of the seq-based strategy, we identified SS-miRNAs in *Arabidopsis thaliana* using two approaches (see Section 4). The results of homology-based strategy produced 104 species-specific candidates from 98 miRNA families, whereas 95 SS-miRNAs from 92 miRNA families were defined as species-specific by the seq-based strategy (Figure 1B). The overlapping results suggested that 21 miRNAs from 18 families defined as false positives and 12 miRNAs from 12 families defined as false negatives of SS-miRNAs were corrected owing to the improvement of the seq-based method (Figure 1B).

Then, we performed two case studies to elaborate on these two drawbacks. As a real SS-miRNA, the sequence of the *MIR771* hairpin in *A. thaliana* was searched against the genome of *Arabidopsis lyrata*, and a high-similarity sequence (referred to as *MIR771*-like) was found (Figure 1C). However, the reads corresponding to the mature sequence of miR771 were not detected using the available sRNA-seq datasets in *A. lyrata*. Furthermore, a previous study supported the 22 nt miR771 in *A. thaliana* as a secondary siRNA trigger [34]. Additionally, the secondary structure of *MIR771*-like showed one-nucleotide variant in the mature sequence leading to only 21 nt in the double-strand arm (Figure 1C). Both findings reveal that the *MIR771*-like in *A. lyrata* was not a bona fide miRNA, supporting the specific *MIR771* in *A. thaliana*. Another example showed a false positive of SS-miRNA caused by the homology-based strategy. As a functional well-studied miRNA family, *MIR398* was proven to be present before the divergence of gymnosperms and angiosperms [35]. However, the comparison of hairpin sequences of the *MIR398* family in *A. lyrata* and *A. thaliana* exhibited low similarity (36.5%) when the sequence similarities were limited to the 20 bp of the mature region, the 6 bp of the star region, and the 4 bp of the loop region (Figure 1D). Taken together, these results demonstrated that the seq-based strategy is a more accurate method for identifying SS-miRNAs in plants.

### 2.2. Identification of SS-miRNAs in 81 Plants

**Figure 2:**
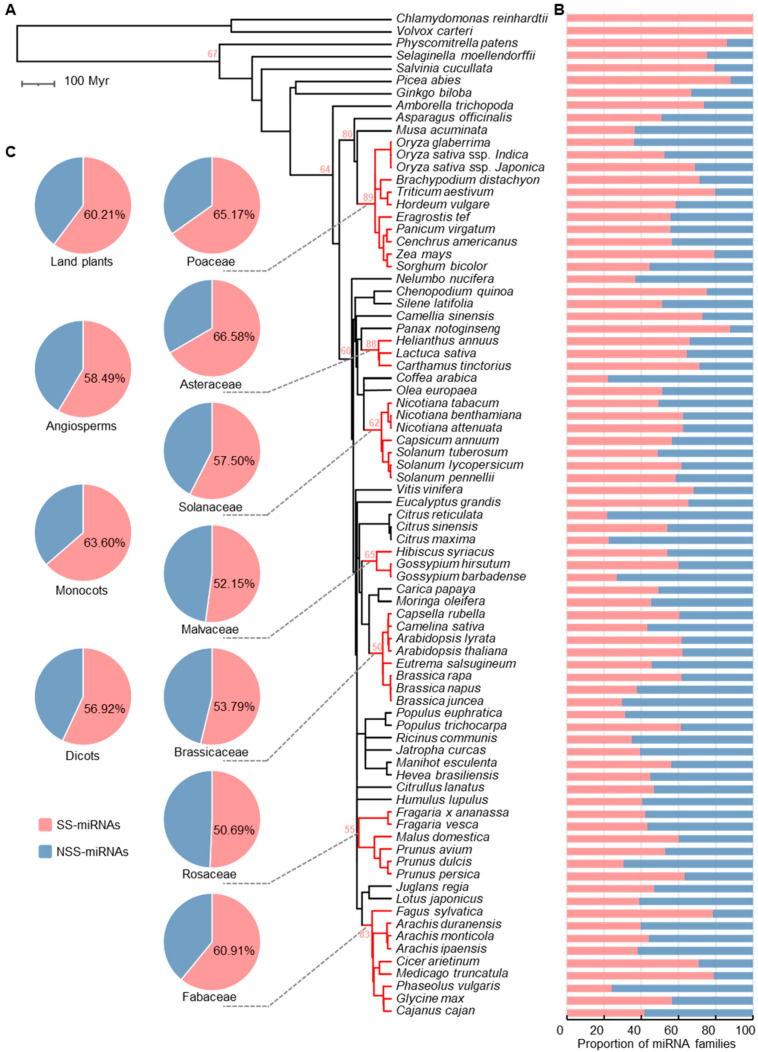
The panorama of SS-miRNA families in land plants. (**A**) A phylogenetic tree of 81 representative plant species retrieved from TimeTree.org. The mean numbers of SS-miRNA families in the taxonomic families are marked on the tree with red text. (**B**) Bar charts showing the proportions of SS-miRNA families and NSS-miRNA families in 81 species. (**C**) Pie charts showing the proportions of SS-miRNA families and NSS-miRNA families in land plants, angiosperms, monocots, dicots, and seven representative families.

As classic bioinformatic tools for miRNA annotation, miRDeep-P [36] and the latest version of miRDeep-P2 [33], based on the newly updated criteria [32], have successfully identified miRNAs in hundreds of plant species [17,37]. The internal algorithm of miRDeep-P2 requires miRNA candidates with plausible hairpin-structured precursors and canonical read distributions. In addition, the recently updated plant miRNA encyclopedia database (PmiREN) provided an opportunity to systemically perform a kingdom-wide survey of SS-miRNAs [17,37]. MiRNAs in PmiREN were identified with a standardized workflow for miRNA identification based on miRDeep-P2 [33] using a variety of accessible sRNA-seq datasets from 179 species. However, some of the species only contained dozens of miRNAs due to the quantity and quality of the sRNA-seq datasets and incomplete genome references, suggesting that the completeness of the miRNA repertoire should be seriously considered for the conservation analysis. Therefore, we selected 2 species of chlorophytes and 79 species that contained seven highly conserved miRNA families of land plants as well as more than one hundred miRNAs for the following analysis. After the quality control, 21,444 high-quality miRNAs belonging to 8978 miRNA families in 81 species phylogenetically ranging from chlorophytes to angiosperms were used to identify SS-miRNAs (Table S1). Using a customized Perl script specifically designed for the seq-based strategy, we identified a total of 8048 miRNAs (37.5%) from 5499 miRNA families (61.2%) as SS-miRNAs (Figure 2A,B and Figure S1).

### 2.3. Diversification of SS-miRNAs Suggests High Turnover of miRNAs

Chlamydomonas reinhardtii and Volvox carteri are two species belonging to chlorophytes who were estimated to have diverged ~220 million years ago (Mya) [38]. We found that none of the 235 identified algal miRNA families were present in land plants (Figure 2B). Moreover, no conserved miRNAs were found between the two species (Figure 2B). Given that only two algae were in our analysis, it was still controversial to determine the conservation of miRNAs in algae.

In land plants, 5264 (60.2%) miRNA families belonging to 79 species were species-specific (Figure 2C). Our results suggested that the proportion of SS-miRNA families in the five nonangiosperm species was higher than that in angiosperms, including one moss (Physcomitrella patens, 115 or 85.8% SS-miRNA families), one lycophyte (Selaginella moellendorffii, 54 or 75.0% SS-miRNA families), one fern (Salvinia cucullata, 50 or 79.4% SS-miRNA families), and two gymnosperms (Picea abies, 234 or 88.0% SS-miRNA families and Ginkgo biloba, 82 or 66.7% SS-miRNA families) (Figure 2B).

Within the examined angiosperm species, 4729 or 58.5% of miRNA families were species-specific (Figure 2C). The proportion of SS-miRNA families in monocots (63.6%) was slightly higher compared with that in dicots (56.9%) (Figure 2C). In the seven taxonomic families of angiosperms, the proportions of SS-miRNA families ranged from 50.7% in Rosaceae to 66.6% in Asteraceae (Figure 2C). Furthermore, the average number of SS-miRNA families in the seven families ranged from 50 to 89 (Figure 2A). At the species level, we found that the proportions of SS-miRNA families varied dramatically, ranging from 21.4% in *Citrus reticulate* to 88.7% in *Panax notoginseng* (Figure 2B). Despite the great differences among species divided over a long period of time, the proportions of SS-miRNA families at the intragenus level were quite similar. Taken together, these findings indicated the high turnover rate of SS-miRNAs, even over short evolutionary distances.

### 2.4. Intrinsic Features of SS-miRNAs

To distinguish the intrinsic features of SS-miRNAs from NSS-miRNAs, we tested four parameters, including the normalized minimal free energy (NMFE) of the hairpins, the adenine–uracil (AU) contents of mature miRNAs and hairpins, the first bases in the 5*′* ends of mature miRNAs, and the lengths of mature miRNAs and hairpins.

**Figure 3:**
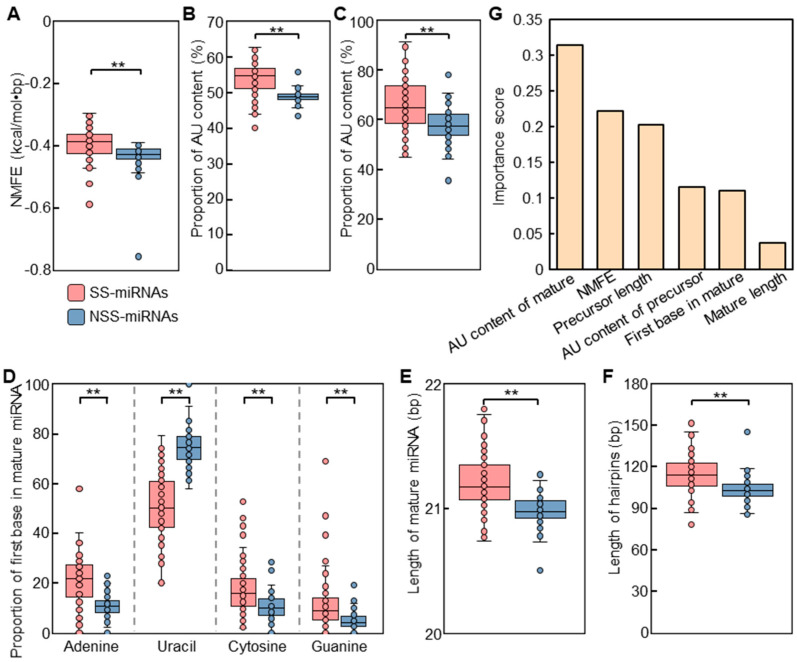
Comparison of features between SS-miRNAs and NSS-miRNAs. (**A**) NMFE of precursor miRNAs between SS-miRNAs and NSS-miRNAs. (**B**,**C**) AU contents of mature miRNAs (**B**) and precursor miRNAs (**C**) between SS-miRNAs and NSS-miRNAs. (**D**) Base composition of the first nucleotides of the 5′ ends of mature miRNAs between SS-miRNAs and NSS-miRNAs. (**E**,**F**) Lengths of mature miRNAs (**E**) and hairpins (**F**) between SS-miRNAs and NSS-miRNAs. ** *p* < 0.01 by independent-samples *t*-test. The SS-miRNAs and NSS-miRNAs are colored in red and blue, respectively. (**G**) Assessment for the relative importance of the 12 examined features by a machine learning approach based on a gradient-boosting decision tree algorithm.

NMFE, reflecting the folding stability of the hairpin structures, appeared to be significantly different between SS-miRNAs and NSS-miRNAs. These findings suggested that the structures of NSS-miRNAs were more stable than those of SS-miRNAs (Figure 3A). Both the mature sequences and hairpins of SS-miRNAs reflected significant higher AU contents than those of NSS-miRNAs (Figure 3B,C, and Figures S2 and S3; Tables S2 and S3). The first bases in the 5*′* ends of the mature sequences of both SS-miRNAs and NSS-miRNAs had a strong bias toward uracil, consistent with the previous results found in plant miRNAs [39] (Figure 3D and Figrue S4; Table S4). Meanwhile, our results also found that the skew to uracil in NSS-miRNAs was significantly higher than that in SS-miRNAs. Analyzing the length of the mature sequences suggested the dominance of 21 nt miRNAs, whereas slightly longer SS-miRNAs was noticed (Figure 3E; Table S5). The lengths of hairpins of SS-miRNAs were significantly longer than those of NSS-miRNAs (Figure 3F; Table S5). These findings indicated that these SS-miRNAs were going through an evolutionary trajectory to the conserved miRNAs with the canonical features.

As these four parameters were significant for discerning the SS-miRNAs from NSS-miRNAs, the importance levels of these parameters were worth determining. Therefore, we performed a machine learning method based on a gradient-boosting decision tree algorithm to assess the relative importance of these parameters. After a simulation process with an overall accuracy of 82%, the results suggested that the AU content of mature miRNAs was the most influential feature (Figure 3G).

### 2.5. SS-miRNAs Lie Close to or within Genes

**Figure 4:**
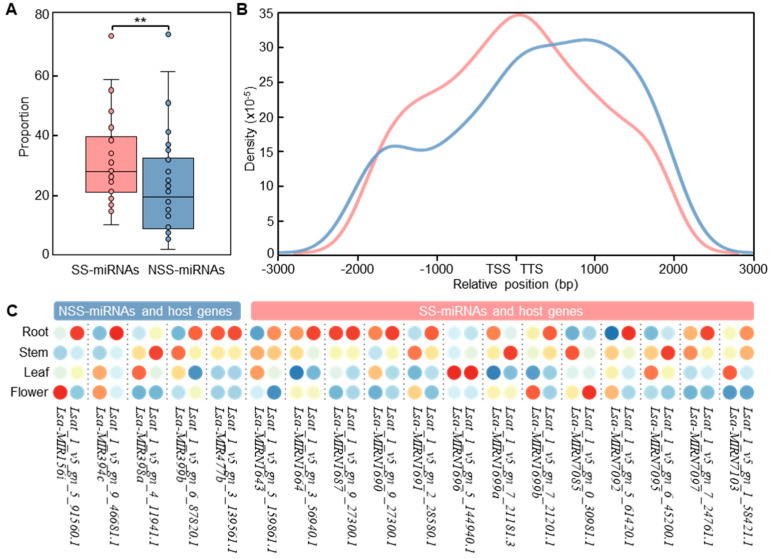
The locations of SS-miRNAs were closed to or within genes. (**A**) A box plot showing the proportion of SS-miRNAs and NSS-miRNAs with overlapped genes (** *p* < 0.01 by independent-samples *t*-test). The SS-miRNAs and NSS-miRNAs are colored in red and blue, respectively. (**B**) Density distribution of SS-miRNAs and NSS-miRNAs close to genes. TSS, transcription start site. TTS, transcription termination site. The SS-miRNAs and NSS-miRNAs are colored in red and blue, respectively. (**C**) The relative expression levels of SS-miRNAs, NSS-miRNAs, and overlapped host genes in four tissues of *L. sativa* are indicated with colors from blue to red. The colored circles from red to blue indicate the relative expression from high to low.

To investigate the relative distribution of SS-miRNAs, we selected 28 phylogenetically representative species with high-quality gene annotations, including two green algae (*V. carteri* and *C. reinhardtii*), one moss (*Physcomitrella patens*), one lycophyte (*Selaginella moellendorffii*), one basally branching angiosperm (*Amborella trichopoda*), four monocots, and nineteen dicotyledons from ten taxonomic families (Table S6). Through intersecting the genomic locations between miRNA hairpins and protein-coding genes, our results suggested that the proportion of SS-miRNAs located within genes was significantly higher than that of NSS-miRNAs (Figure 4A). Moreover, we found that SS-miRNAs were closer to genes compared with NSS-miRNAs (Figure 4B). The proportion of SS-miRNAs located upstream of transcription start sites was slightly higher than that of NSS-miRNAs. However, the position of NSS-miRNAs had a significant bias toward being downstream of transcription termination sites. Our results indicated that SS-miRNAs lie close to or within genes significantly more than NSS-miRNAs.

Several studies assumed that miRNAs localized in the vicinity of genes shared promoters with their host genes [40,41]. However, this option was still controversial owing to opposite observations [42,43,44]. To test this hypothesis, we used synchronous and high-quality sRNA-seq and RNA-seq datasets in four tissues of *Lactuca sativa* sequenced by our group [45]. Employing 5 overlapped NSS-miRNAs and 13 SS-miRNAs in *L. sativa*, we scanned the expression levels of these miRNAs from sRNA-seq and host genes from RNA-seq in the root, stem, leaf, and flower. The results suggested that only one NSS-miRNA (*Lsa-MIR477b*) and four SS-miRNAs (*Lsa-MIRN1664*, *Lsa-MIRN1687*, *Lsa-MIRN1690*, and *Lsa-MIRN1696*) were highly expressed in the same tissues as their corresponding host genes (Figure 4C). In addition, a correlation analysis also supported the distinct expression pattern for both SS-miRNAs (Pearson’s *r* = −0.07, *p* = 0.77) and NSS-miRNAs (Pearson’s *r* = 0.53, *p* < 0.01), suggesting that the hypothesis of cotranscription was still dubious.

### 2.6. Association of SS-miRNA Target Genes with Metabolism

**Figure 5:**
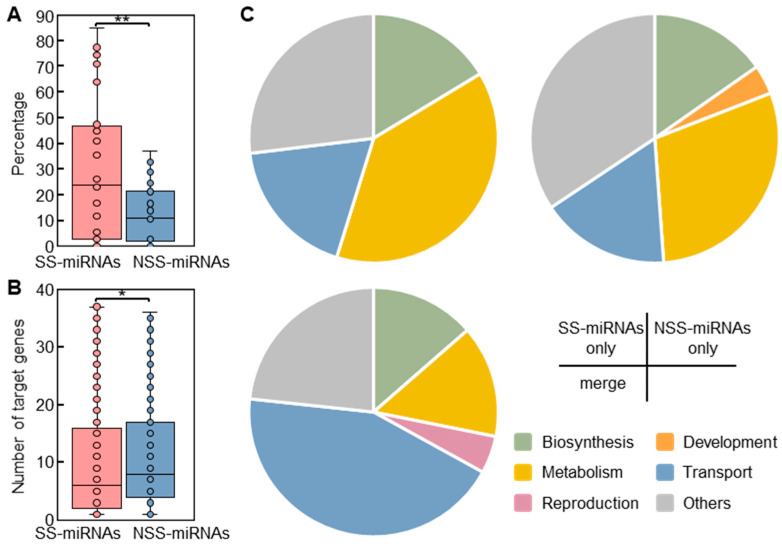
A comparison of the spectrum of target genes of SS-miRNAs and NSS-miRNAs. (**A**) A box plot showing the proportion of SS-miRNAs and NSS-miRNAs without predicted target genes (** *p* < 0.01 by independent-samples *t*-test). (**B**) The number of predicted target genes of SS-miRNAs and NSS-miRNAs (* *p* < 0.05 by independent-samples *t*-test). The SS-miRNAs and NSS-miRNAs are colored in red and blue, respectively. (**C**) Pie charts showing the proportions of enriched GO terms associated with target genes of SS-miRNAs, NSS-miRNAs, and both.

Using an in silico approach, we identified 125,267 putative target genes for 7966 miRNAs in the 28 examined plants with high-quality gene annotation. Our results suggested that the mean proportion of SS-miRNAs (20.7%) without a predicted target gene was significantly higher than that of NSS-miRNAs (9.7%) (Figure 5A). Contrary to a previous hypothesis that novel miRNAs possess more target genes that appear at random in the genome [46], we observed that SS-miRNAs regulated fewer target genes than NSS-miRNAs (Figure 5B). These findings implied that new miRNAs in plants were subjected to stronger selection than previously thought. A gene ontology (GO) analysis revealed that the molecular functions of the SS-miRNA target genes, compared to those of the NSS-miRNAs, were more enriched, with 40 terms related to metabolism (Figure 5C; Tables S7–S9).

Our results indicated that SS-miRNAs frequently regulated diversified metabolic processes in plants.

## 3. Discussion

Genomic innovation is one of the crucial factors for multicellular plants to produce a vast array of metabolites, thereby adapting to a multitude of environments [47,48]. Species-specific genes as well as noncoding RNAs are observed to contribute to species-specific adaptations [6,49,50,51]. However, two major stumbling blocks impeded the global and convincing identification of SS-miRNAs. In this study, we implemented a comprehensive investigation of SS-miRNAs from 81 plant species across algae and land plants using the seq-based strategy, thereby providing the first large-scale repository of SS-miRNAs (Figure 1 and Figure 2). Moreover, we realized the definition of SS-miRNAs is affected by sampling and phylogenetic diversity, and further analysis should provide more accurate annotation once miRNAs are identified in a broader range of species.

In this study, a high turnover rate for miRNAs in plants was observed. Our findings indicated that SS-miRNA families account for 60.2% of miRNA families in land plants as well as 58.5% in angiosperms (Figure 2B,C). In the seven representative families, over half of miRNA families were present in only one examined species. These results indicated the rapid gain and loss of miRNAs in plants. Several possible modes describing the origination of new miRNAs have been proposed [10,21,22,23,24,52] (Figure S5), including a recent study that suggested that MITEs are the predominant genomic source of new miRNAs in angiosperms [53]. We found that SS-miRNAs are AU-rich sequences compared with NSS-miRNAs (Figure 3B,C), which is consistent with the sequence characteristics of MITEs [54]. The model constructed by machine learning also suggested that the AU content of mature miRNA was the major difference between SS-miRNAs and NSS-miRNAs (Figure 3G). However, the loss of miRNAs is still poorly understood but is necessary, as the generation and loss of miRNAs are two sides of the same coin (Figure S5).

We also noticed two results that contradict previous studies. First, SS-miRNAs and their corresponding host genes presented an inconsistent expression pattern in four tissues of *L. sativa* (Figure 4C), questioning the cotranscription of novel miRNAs and genes [40,41]. Second, our findings suggested that the proportion of SS-miRNAs without predicted target genes was more than double that of NSS-miRNAs (Figure 5A). Moreover, the mean number of target genes of SS-miRNAs was less than that of NSS-miRNAs (Figure 5B). Our results imply that SS-miRNAs were subjected to more stringent selection than previous thought [46].

## 4. Materials and Methods

### 4.1. SS-miRNA Identification Using Seq-Based Strategy

MiRNA sequences and annotation information of 81 species were downloaded from the PmiREN2.0 database (https://www.pmiren.com, accessed on 1 July 2022) [37], where stored high-quality identified miRNAs were required to have more than 75% sRNA-seq reads in the mature and star miRNA regions and no more than 20% read overlap in the mature and star regions. Based on this strategy, we could discern miRNAs from siRNAs. Then, we performed a built-in Perl script in miRDeep-P2, merge_and_rank.bash, to group the miRNA families. The default parameter of this script was no more than two mismatches in the mature miRNA among the members of a family, as recommended previously [37]. Finally, a reciprocal comparison among the miRNA repositories of 81 species was implemented. MiRNA families detected in only one species were defined as species-specific.

### 4.2. SS-miRNA Identification in A. thaliana and A. lyrata Using Homology-Based Strategy

Genome sequences of A. thaliana and A. lyrata were downloaded from Phytozome v13 (https://phytozome-next.jgi.doe.gov/, accessed on 1 July 2022; Table S6) [55]. Sequences of hairpin miRNAs in A. thaliana and A. lyrata were retrieved from the PmiREN2.0 database (https://www.pmiren.com, accessed on 1 July 2022) [37]. For the homology-based strategy, sequences of miRNA hairpins from one species were used to search against the genomes from the other species with BLAST [56] with an e-value < 1 × 10*^−^*^10^. The miRNAs that contained less than 70% sequences matched to hits were filtered out and considered as final results. MiRNAs that were absent from the final results were defined as species-specific in *A. thaliana* or *A. lyrata*.

### 4.3. XGBoost-Based Machine Learning Model

In this study, four features of SS-miRNAs and NSS-miRNAs were calculated as the input datasets for machine learning, including the normalized minimal free energy of the hairpins, the adenine–uracil contents of mature miRNAs and hairpins, the first bases in the 5*′* ends of mature miRNAs, and lengths of mature miRNAs and hairpins. Then, a gradient-boosting decision tree implemented in XGBoost (Extreme Gradient Boosting) [57] was used to construct a classifying model and evaluate the importance of these features. The parameters of the learning rate were set as 1, and the maximum depth was set as 2. The objective function was set as “binary::logistic”. The boosting iteration number was set as 50. The “xgb.importance” function in XGBoost was used to calculated and rank the importance scores of the four features.

### 4.4. Target Gene Prediction

In this study, target genes of miRNAs were predicted by a common bioinformatic method, psRNAtarget [58]. The mature miRNA sequences and mRNA transcripts of the corresponding species were uploaded to the psRNATarget webserver. The newest default parameters of Schema V2 (2017 release) were used, except that the default expectation threshold of 5 was reduced to a more restricted value of 3.

### 4.5. Relative Distribution of miRNAs

The genome coordinates of miRNA hairpins were retrieved from PmiREN2.0 [37]. Genome annotation information of 28 species is shown in Table S6. Then, bedtools was implemented to intersect the coordinates of each miRNA hairpin and protein-coding genes according to the annotation. An in-house Perl script was performed to search for miRNAs flanking protein-coding genes.

### 4.6. GO Enrichment Analysis

Annotations of GO terms were obtained from Phytozome v13 (https://phytozome-next.jgi.doe.gov/, accessed on 1 July 2022). The associations of the predicted target genes of SS-miRNAs and NSS-miRNAs with the GO terms were analyzed using custom scripts. Fisher’s exact test with the Benjamini–Hochberg correction was used to find enriched GO terms with the adjusted *p* value set as 0.05.

### 4.7. Other Statistical Analyses

If not stated specifically, the comparison of two distributions of values was tested with a paired-samples *t*-test (two-tailed). *p* values are shown as exact values or otherwise referenced with a symbol according to the following scales: * *p* < 0.05; ** *p* < 0.01.

## Data Availability

All code used in this study is freely accessible via the GitHub repository (https://github.com/little-raccoon/SS-miRNA/). Supplementary Data are available at International Journal of Molecular Sciences online.

## Supplementary Materials

The following supporting information can be downloaded at: https://www.mdpi.com/article/10.3390/ijms232214273/s1.
