# Supplementary material for: Identification of Species-Specific MicroRNAs Provides Insights into Dynamic Evolution of MicroRNAs in Plants
Source: Int J Mol Sci. 2022 Nov 17;23(22):14273. doi: 10.3390/ijms232214273 (PMC9698635; doi:10.3390/ijms232214273)
Supplement: Supplementary file 1 [file ijms-23-14273-s001.zip › SS-miRNA_S Figures_S1-S5.pdf]

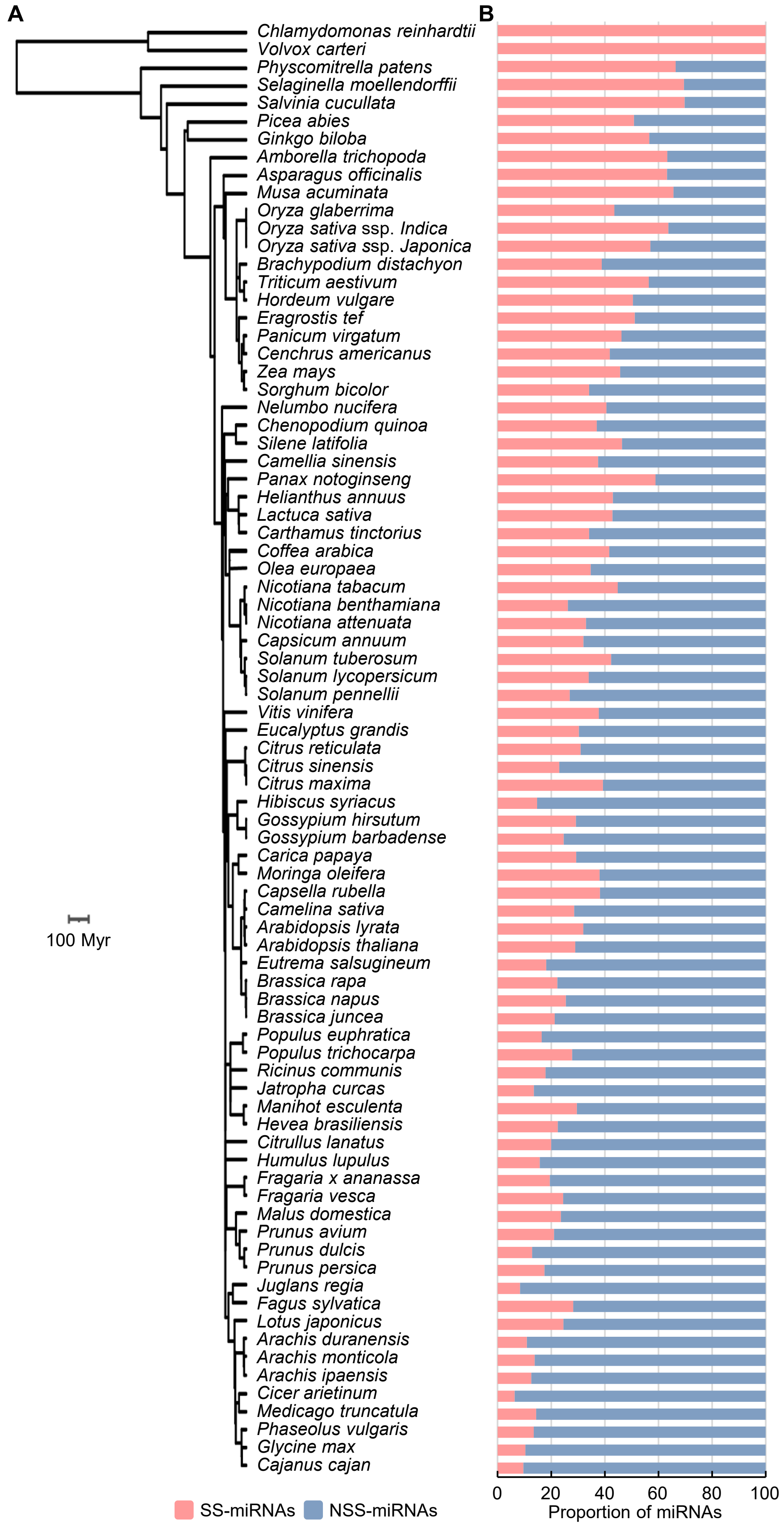

Figure S1. A panorama of SS-miRNA and NSS-miRNA in 81 species.
A. Phylogenetic tree of 81 representative plant species retrieved from TimeTree.org. **B.** Bar graphs showing proportions of SS-miRNAs and NSS-miRNAs in given species.

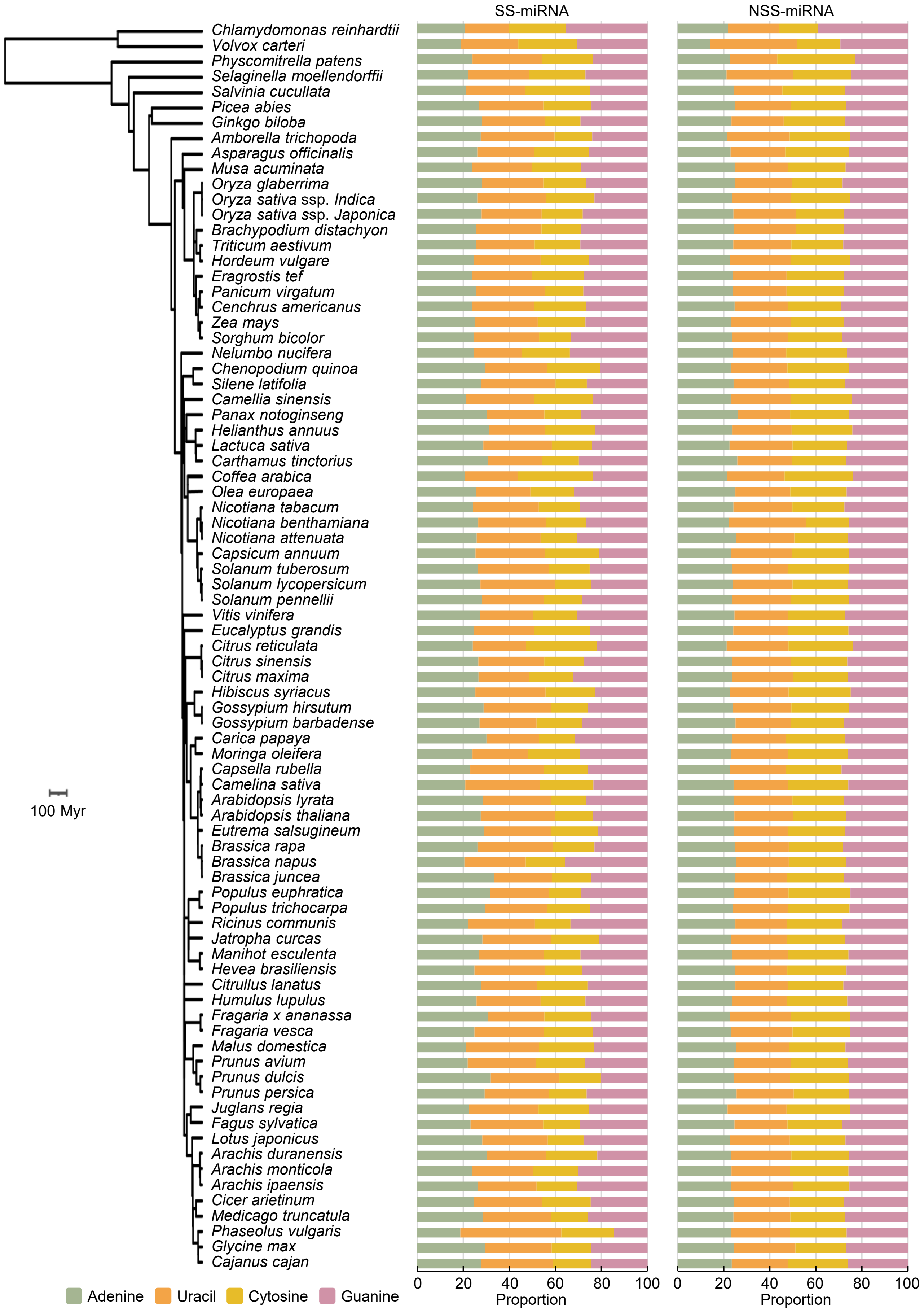

Figure S2. Base composition of mature SS-miRNAs and NSS-miRNAs in 81 species.

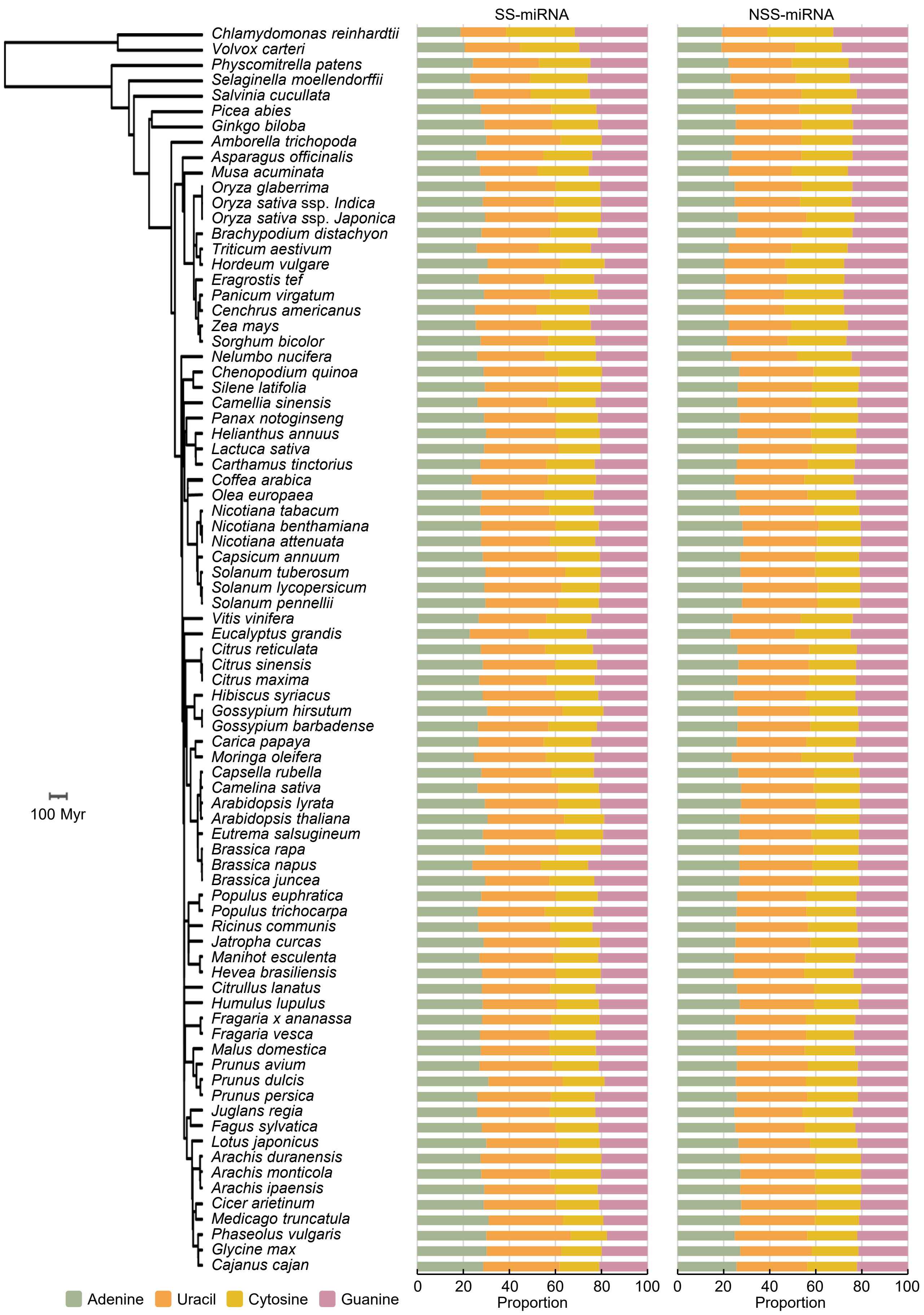

Figure S3. Base composition of hairpins of SS-miRNAs and NSS-miRNAs in 81 species.

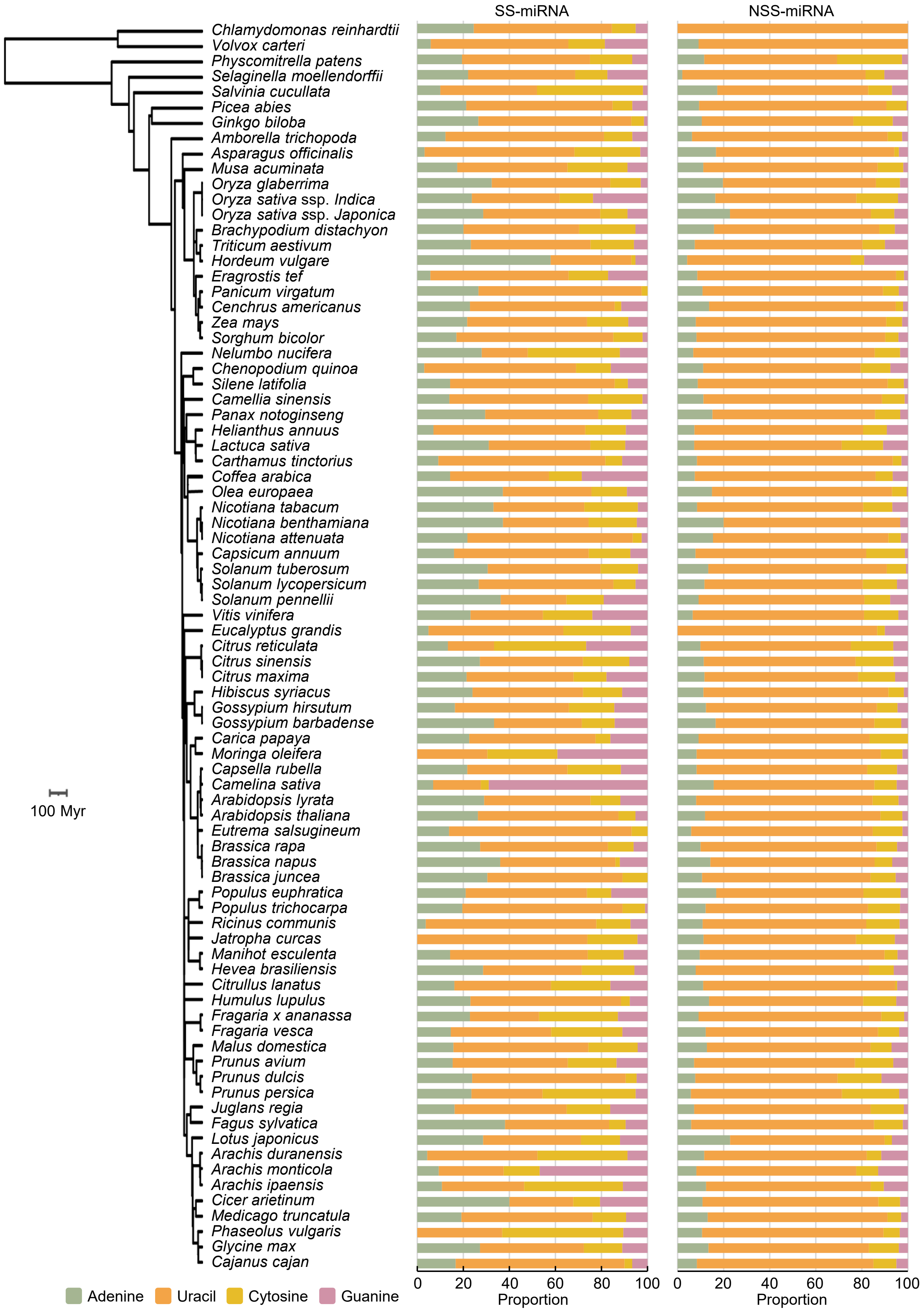

Figure S4. Base composition of first base in 5' of mature SS-miRNAs and NSS-miRNAs in 81 species.

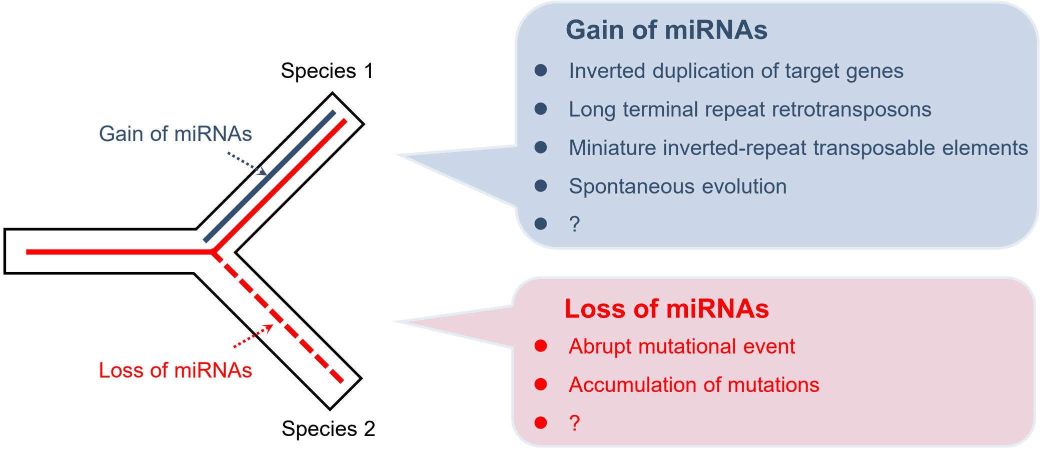

Figure S5. Evolutionary model of SS-miRNAs.
